# PKM2 Expression as Biomarker for Resistance to Oxaliplatin-Based Chemotherapy in Colorectal Cancer

**DOI:** 10.3390/cancers12082058

**Published:** 2020-07-25

**Authors:** Maria Sfakianaki, Chara Papadaki, Maria Tzardi, Maria Trypaki, Stavroula Manolakou, Ippokratis Messaritakis, Zenia Saridaki, Elias Athanasakis, Dimitrios Mavroudis, John Tsiaoussis, Nikolaos Gouvas, John Souglakos

**Affiliations:** 1 Laboratory of Translational Oncology, School of Medicine, University of Crete, 71003 Heraklion, Greece; chapapadak@uoc.gr (C.P.); tr.maria@gmail.com (M.T.); sdmanolakou@hotmail.com (S.M.); i_messaritakis@yahoo.com (I.M.); zeniasar@gmail.com (Z.S.); mavroudis@uoc.gr (D.M.); johnsougl@gmail.com (J.S.); 2 Department of Pathology, University General Hospital of Heraklion, 71110 Heraklion, Greece; tzardi@uoc.gr; 3 Department of Surgery, University General Hospital of Heraklion, 71110 Heraklion, Greece; eliasathanasakis@yahoo.gr; 4 Department of Medical Oncology, University General Hospital of Heraklion, 71110 Crete, Greece; 5 Laboratory of Anatomy, School of Medicine, University of Crete, 71003 Heraklion, Greece; tsiaoussis@uoc.gr; 6 School of Medicine, University of Cyprus, Nicosia 1678, Cyprus; nikos.gouvas@gmail.com

**Keywords:** PKM2, *ΚRAS*, *BRAF*, MSI, prediction, colon cancer

## Abstract

The purpose of the current study is to investigate the prognostic significance of M2 isoform of pyruvate kinase (PKM2) *mRNA* expression loss in patients with operable colon cancer (CC). Two hundred sixty-two specimens from patients with stage-III or high-risk stage-II CC (group-A) treated with adjuvant fluoropyrimidine and oxaliplatin chemotherapy (FOLFOX), 118 specimens from metastatic CC patients (group-B) treated with FOLFOX, and 104 metastatic CC patients (group-C) treated with irinotecan-based chemotherapy were analyzed for *PKM2, TS, ERCC1, MYC*, and *NEDD9* mRNA expression, as well as *KRAS* exon2 and *BRAF^V600E^* mutations. High *PKM2* mRNA expression was correlated with left-sided located primaries (*p* = 0.001, group-A; *p* = 0.003, group-B; *p* = 0.001, group-C), high-grade tumors (*p* = 0.001, group-A; *p* = 0.017, group-B; *p* = 0.021, group-C), microsatellite-stable tumors (*p* < 0.001, group-A), pericolic lymph nodes involvement (*p* = 0.018, group-A), and *cMYC* mRNA expression (*p* = 0.002, group-A; *p* = 0.008, group-B; *p* = 0.006, group-C). High *PKM2* mRNA expression was correlated with significantly lower disease free survival (DFS) (*p* = 0.002) and overall survival (OS) (*p* = 0.001) in the group-A. Similarly, *PKM2* mRNA expression was associated with significantly decreased progression free survival (PFS) (*p* = 0.001) and OS (*p* = 0.001) in group-B. On the contrary, no significant association for the *PKM2* mRNA expression has been observed with either PFS (*p* = 0.612) or OS (*p* = 0.517) in group-C. To conclude, the current study provides evidence for the prediction of *PKM2* mRNA expression oxaliplatin-based treatment resistance.

## 1. Introduction

Colorectal cancer (CRC) represents 9% of all malignant tumors in adults [1]. Even though curative surgical resection shows potential in 70–80% of colon cancer (CC) patients at diagnosis, nearly half of them will develop local or/and metastatic recurrence and will pass away from the disease [2] (GLOBOCAN). Approximately 50% of the patients with stage-III or high-risk stage-II disease could be treated with surgery alone and adjuvant chemotherapy may prevent relapse in another 20–25% of patients [3]. Adjuvant chemotherapy with a combination of a fluoropyrimidine (FP) with oxaliplatin (LOHP) is recommended for stage-III or high risk of relapse stage-II colon patients [3]. CAPOX (capecitabine + oxaliplatin) or FOLFOX are also among the standard treatment options for metastatic disease [3].

The genetic CRC underpinnings are extremely well studied [4] and a multistep process for the carcinogenesis in the colon epithelium, from normal mucosa to invasive cancer, has been suggested more than twenty years ago [5]. Ideally, molecular characteristics acquired from the primary tumors should guide therapeutic decisions and allow medical oncologists to select patients more successfully for the most beneficial and least toxic treatment strategies. At present, microsatellite instability (MSI) status is the only biological signature available in daily clinical practice [6]; however, a surrogate biomarker for treatment efficacy is needed.

Tumor cells favor glycolysis and little pyruvate is dispatched to mitochondria for oxidative phosphorylation even in the presence of sufficient oxygen [7,8]. Aerobic glycolysis provides dividing cells both energy and glycolytic intermediates, which are vital as precursors for amino acids, nucleic acids, and lipids synthesis. Furthermore, acidification of the extracellular microenvironment because of the expanded pyruvate production may facilitate tumor cell invasion and metastasis [9]. One of the most established key regulators of aerobic glycolysis is the embryonic M2 isoform of pyruvate kinase (PKM2), expressed during embryonic development and tumor formation. PKM2 catalyzes the last step of glycolysis by the formation of pyruvate and ATP from phosphoenolpyruvate (PEP) and ADP [10,11]. Recent studies have shown that PKM2 has a bi-functional role within tumors; it exists as a dimeric form with a low PEP affinity and shifts between a tetrameric form that has a high affinity for its substrate PEP [12]. Except for its well-known function in glycolysis, PKM2 also regulates other cellular functions, such as gene transcription and cell cycle progression [13,14]. However, the process of PKM2 effecting CC and the correlation between PKM2 expression levels and CC remains unclear.

Additionally, recent relevant studies proposed that PKM2 expression may be a predictive biomarker of platinum sensitivity in various cancers, including CRC [15,16,17]. In the present study, we investigated the prognostic and predictive value of *PKM2* mRNA expression in primary CC in three different patients’ groups: (A) stage II or III CC treated with adjuvant oxalipaltin and fluoropyrimidine; (B) metastatic CRC (mCRC) treated with first-line treatment, oxalipaltin, and fluoropyrimidine; and (C) mCRC treated with first-line treatment, irinotecan, and fluoropyrimidine (FOLFIRI regimen), as the control group. The *PKM2* mRNA expression was also analyzed in adenomas with high and low dysplasia. These findings may provide evidence of *PKM2* mRNA expression prospective role as a predictive and prognostic biomarker in CC.

## 2. Results

### 2.1. Patients’ Characteristics and Clinico-Pathological Features

The main demographic and clinical characteristics of the study population are summarized in Table 1 for patients with stage II–III (group-A) and mCRC (groups-B/C), respectively. Briefly, group-A patients that received postoperative combination (fluoropyrimide and oxaliplatin) adjuvant chemotherapy (CAPOX or FOLFOX) were predominantly males (58%) with a median age of 67 years; 65% of the patients had a primary tumor located in the left colon and 60% were diagnosed with stage III CC (Table 1). At the time of analysis and after a median follow-up of 120.7 months (min–max: 11.3–161.1 months); 71 (27%) disease relapses and 48 (18%) deaths were recorded.

Likewise, group-B patients (treated with oxaliplatin-based chemotherapy) were predominantly male (64%), with a median age of 65 years, with the vast majority with ECOG PS 0–1 (93%) and primary tumor located principally in the left colon in 63% of the cases; the median number of involved organs was 1 (range 1–4), while *KRAS* exon 2 or *BRAF^V600E^* mutations were found in 36 and 7%, respectively (Table 1). At the time of analysis and after a median follow-up of 49.3 months (min–max: 2.4–167.3 months), 115 (96%) disease relapses and 114 (97%) deaths were recorded in the validation group.

Finally, group-C patients (treated with irinotecan-based treatment) presented similar characteristics with those in the validation group: median age 65 years, 64% males, 75% left-sided primary tumors, median number of metastatic sites 1, *KRAS* exon 2 34%, and *BRAF^V600E^* mutations 6%. After a median follow-up of 47.4 months (min–max: 1.8–155.7 months), 94 (90%) disease relapses and 91 (88%) deaths were recorded in the control group. In addition, *PKM2* mRNA expression was evaluated in 24 adenomas with high-grade dysplasia, 18 adenomas with low-grade dysplasia, and 50 specimens from normal colonic mucosa.

### 2.2. *PKM2* mRNA Expression in Different CRC groups, Adenomas, and Normal Mucosa

*PKM2* mRNA expression was successfully analyzed in 258 out of the 262 specimens from group-A, in all 118 specimens from group-B patients, and in all 104 samples in group-C. Moreover, *PKM2* was successfully amplified in all specimens from adenomas and normal mucosa. No difference in *PKM2* mRNA expression was observed among the three groups. The median *PKM2* mRNA expression for the whole group of patients with CC was 15.11 (min–max: 1.61–97.55); more specifically, 15.11 (min–max: 3.39–41.59) for group-A, 15.24 (min–max: 1.71–97.55) for group-B, and 16.24 (min–max: 1.61–45.00) for group-C (all *p* values > 0.05). In contrast, *PKM2* mRNA expression was significantly lower in normal mucosa (5.16; min–max: 0.1–11.12) in comparison with that in CC specimens (*p* < 0.001). Similarly, *PKM2* mRNA expression was significantly lower in adenomas with low-grade dysplasia (8.04; min–max: 0.1–15.79) in comparison with that in tumoral specimens (*p* = 0.002). No significant difference in *PKM2* mRNA expression was observed between adenomas with high-grade dysplasia (14.57; min–max: 0.92–15.79) and CC samples (*p* = 0. 287).

### 2.3. *PKM2* mRNA Expression in Different Groups and Correlations with Clinico-Pathological Features and Analysed Markers

In the total number of tumour samples from groups A, B, and C, *PKM2* mRNA expression was significantly correlated with *MYC* mRNA expression (ρ^2^ = 0.153, *p* = 0.004). The same finding was observed when each group was analyzed separately for *PKM2* and *MYC* mRNA expression (ρ^2^ = 0.236, *p* = 0.002, for group-A; ρ^2^ = 0.112, *p* = 0.008, for group-B; ρ^2^ = 0.178, *p* = 0.006, for group-C).

In addition, high *PKM2* mRNA expression was associated with left-sided tumors in all patients (group-A: *p* = 0.011, group-B: *p* = 0.003, and group-C: *p* = 0.001) and high grade (undifferentiated) tumors in all three groups (*p* = 0.001, *p* = 0.017, and *p* = 0.021 for groups A, B, and C, respectively; Table 2). Furthermore, high *PKM2* mRNA expression was significantly correlated with *KRAS* exon2 and *BRAF^V600E^* mutations, in patients with mCRC (*p* = 0.009, *p* = 0.026 for *KRAS* exon2 mutations and *p* = 0.041, *p* = 0.05 for *BRAF^V600E^* mutations in groups B and C, respectively), but not in group-A (*p* = 0.870 and *p* = 0.109 for *KRAS* exon2 and *BRAF^V600E^* mutations, respectively; Table 2). Finally, high *PKM2* mRNA expression was significantly recorded in tumors with proficient MMR (*p* < 0.001), mucinous features (*p* = 0.001), and those with infiltrated regional lymph nodes (*p* = 0.018) in group-A, whereas these parameters were not available in groups B or C (Table 2).

### 2.4. Patients’ Outcome According to PKM2 mRNA Expression

**Figure 1:**
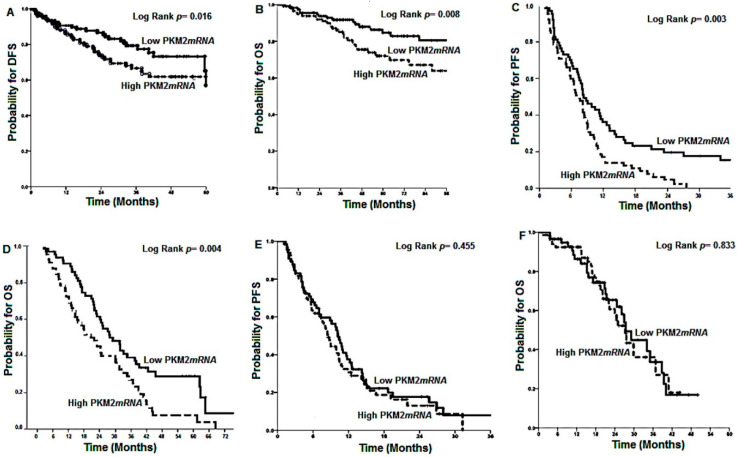
Kaplan Meier analysis in group A, group B, and group C colorectal cancer (CRC) according to M2 isoform of pyruvate kinase (PKM2) mRNA expression. (**A**) Five-year disease free survival (DFS) rate according to *PKM2* mRNA expression in patients with stage II or III colon cancer treated with FOLFOX or CAPOX; (**B**) five-year overall survival (OS) rate according to *PKM2* mRNA expression in patients with stage II or III colon cancer treated with FOLFOX or CAPOX; (**C**) progression free survival (PFS) according to *PKM2* mRNA expression in patients with mCRC treated with oxaliplatin-based first-line treatment; (**D**) median OS according to *PKM2* mRNA expression in patients with m CRC treated with oxaliplatin-based first-line treatment; (**E**) PFS according to *PKM2* mRNA expression in patients with mCRC treated with irinotecan-based first-line treatment; (**F**) median OS according to *PKM2* mRNA expression in patients with mCRC treated with irinotecan-based first-line treatment.

In group-A, tumors with high *PKM2* mRNA expression were significantly associated with a lower five-year DFS rate as compared with low PKM2 mRNA expression levels (68.6% vs. 77.9%, respectively; *p* = 0.016) (Figure 1A) and lower five-year survival rates (75.2 vs. 86.1%, for high and low *PKM2* mRNA expression, respectively; *p* = 0.008; Figure 1B). Similarly, in group-B, tumours with high *PKM2* mRNA expression presented significantly lower PFS (6.7 months, 95% confidence interval (CI): 4.8–7.5 months) in comparison with those with low *PKM2* mRNA expression tumours (9.1 months, 95% CI: 7.7–11.2 months; *p* = 0.003; Figure 1C). Similarly, shorter median OS was significantly correlated with high *PKM2* mRNA expression (21.9 months (95% CI: 16.0–24.7) versus 30.2 months (95% CI: 24.0–37.3) for high *PKM2* mRNA expression, respectively; *p* = 0.004; Figure 1D) in the same patients’ group-B. In contrast, in group-C, no significant difference was observed among patients with high compared to low PKM2 mRNA expression in either PFS [8.4 months (95% CI: 7.2–9.8) vs. 10.2 months (95% CI: 8.4–11.9; *p* = 0.445, respectively] (Figure 1E) or median OS [27.3 months (95% CI: 23.1–31.4) vs. 27.5 months (95% CI: 23.4–31.6); *p* = 0.883, respectively] (Figure 1F).

### 2.5. Multivariate and Univariate Analysis

Table 3 summarizes the univariate analysis for DFS (group-A) or PFS (group-B) and median OS, respectively. Univariate analysis demonstrated that high *PKM2* mRNA expression was significantly correlated with decreased DFS in group-A (hazard ratio (HR): 1.82, 95% CI: 1.21–2.96; *p* = 0.003) and decreased PFS in group-B (HR: 1.91, 95% CI: 1.34–2.99; *p* = 0.002), but not in group-C (HR: 1.06, 95% CI: 0.65–1.31; *p* = 0.769) (Table 3). Similarly, high *PKM2* mRNA levels were significantly associated with shorter median OS in group-A (HR: 1.84, 95% CI: 1.29–3.26; *p* = 0.002) and in group-B (HR: 2.12, 95% CI: 1.51–3.17; *p* = 0.002), but not in group-C (HR: 1.04, 95% CI: 0.58–1.24; *p* = 0.811 (Table 3).

Furthermore, Table 4 shows the results of the multivariate analysis for median for DFS (group-A) or PFS (group-B) and median OS, respectively. Specifically, multivariate analysis revealed high *PKM2* mRNA levels as an independent factor for decreased DFS (HR: 1.88, 95% CI: 1.37–2.99; *p* = 0.002) and median OS (HR: 1.99, 95% CI: 1.45–2.97; *p* = 0.001) in group-B (Table 4). Likewise, multivariate analysis shows high *PKM2* mRNA expression as an independent factor for decreased PFS (HR: 1.94, 95% CI: 1.38–3.32; *p* = 0.001) and median OS (HR: 1.94, 95% CI: 1.38–3.32; *p* = 0.001) in group-A (Table 4). In contrast, no significant effect of high *PKM2* mRNA expression was observed in either PFS or median OS in group-C.

## 3. Discussion

PKM2 is associated with aerobic glycolysis and cell growth in various tumors, but the pattern of *PKM2* in CRC remains unclear [10]. In the present study, we examined the role of tumoural *PKM2* mRNA expression as a predictive biomarker in the outcome of stage-II/IIICC or mCRC patients. Patients with operable CC treated with FOLFOX (group-A) with high *PKM2* mRNA expression presented significantly lower survival rates. These data were validated in an independent cohort of mCRC patients treated with FOLFOX (group-B). On the contrary, in the control group of patients treated with FOLFIRI in the first-line setting (group-C), no significant correlation of high *PKM2* mRNA levels was observed to OS rates. *BRAF^V600E^* was an independent prognostic factor for OS in all three patients groups, while *KRAS* was not in any of the three groups. Taking into account that no significant effect of high PKM2 expression presented in patients who did not receive oxaliplatin, PKM2 could be examined only as a predictive factor for oxaliplatin-based treatment.

PKM2 is universally expressed in all tissues throughout the embryonic division, in normal proliferating cells, and in the different tissues such as fat and lung tissue and especially tumor, which suggests that the capacity to balance pyruvate kinase enzymatic activity is significant in actively proliferating cells [12]. Christofk et al. showed that *PKM2* expression is necessary for aerobic glycolysis and that this metabolic phenotype provides a selective growth advantage for tumor cells in vivo [7].

Oxaliplatin resistance gain is a complex mechanism mainly based on alteration of genes and pathways involved in its mechanism of action [18]. It is previously well described that, like other glycolytic enzymes, PKM2 nuclear translocation is plentiful and crucial for the induction of apoptosis under regimen with somatostatin analogues or DNA-damaging agents [19,20]. As oxaliplatin is a DNA-damaging agent, similar mechanisms probably occur after exposure to this drug. Proteomic analysis showed that high PKM2 was associated with higher response rates in oxaliplatin-resistant colorectal cell line [19].

In contrast, other studies presented downregulation of PKM2 protein in both cisplatin-resistant ovarian cancer and human gastrinoma cell lines, respectively [21,22]. In addition, a recent study on bladder cancer tissues revealed that PKM2 inhibition via shRNA or chemical inhibitors provoked increased cisplatin-sensitivity, and thus cell apoptosis [23,24]. Moreover, nuclear PKM2 expression administered an important prediction for the poor prognosis of patients with esophageal squamous cell carcinoma [25,26]. In concordance, Zhu et al. suggested that PKM2 enhances chemosensitivity to cisplatin, through its interaction with the mTOR signaling pathway in cervical cancer [11]. Moreover, in vitro studies identified PKM2 silencing using specific siRNAs as a supposed oxaliplatin-resistance agent in HT29 CRC cell lines, while strikingly, in HCT116 (a p53 wild type cell line), PKM2 silencing significantly increased sensitivity to Oxaliplatin [27].

PKM2 levels in fecal samples were found to be increased with the adenoma–carcinoma progression. In patients with dysplastic polyps, fecal PKM2 levels were higher in those with larger polyps when compared with those with smaller polyps or healthy controls [28]. Except for fecal and serum, PKM2 levels may also be beneficial in distinguishing malignant and benign lesions of the colon or normal controls [29]. Similarly, in the present study, *PKM2* mRNA expression levels were evaluated successfully in three different sample groups, from adenoma to normal colon mucosa and CC mucosa specimens. In particular, our findings clearly revealed statistically significant lower levels in *PKM2* expression in adenoma with low-grade dysplasia and in normal colon mucosa, in comparison with high-grade dysplastic adenomas or that in CC specimens. On the basis of the literature, left-sided CC tumors were associated with better clinical outcome and represent an early-stage disease, decreased tumor size, and well-differentiated tumors [30]. However, our results showed that high *PKM2* mRNA expression associated with left-sided tumors in all patients’ groups and undifferentiated tumors in all three groups, respectively.

In consideration of the principal role of *PKM2* in CRC growth depending on evidence from previous studies [8,10,29], we investigated the interaction of PKM2 expression with known clinic-pathological features, MSI status, and *KRAS* exon 2 and *BRAF^V600E^* mutations, as well as *ERCC1, cMYC, NEDD9*, and *TS* mRNA expression. To our best of knowledge, this is the first research that associates a combination of all these parameters; thus, the outcome of our analysis could possibly serve as a beneficial guide for the everyday clinical practice.

Recent studies have shown that PKM2 also periodically translocate to the nucleus and oversee cell cycle regulator and oncogene expression (in particular, *KRAS* and *cMYC*) [31,32]. Others demonstrated that PKM2 interacts with PI3K/AKT/mTOR and Ras-MAPK pathways with high affinity [33,34]. The present study is in partial agreement with previous evidence, as *BRAF^V600E^* and *KRAS* mutations have been significantly correlated with high *PKM2* mRNA expression only in patients with mCRC (groups B and C), but not in those with stage II–III adjuvant CC (group-A). Last, but not least, the mutational rates of KRAS, BRAF, and Mismatch Repair System (MMR) status demonstrated in the current study are in total agreement with the current scientific bibliography.

Moreover, in agreement with previous studies, our results clearly demonstrate a strong positive correlation between PKM2 and *c-MYC* mRNA expression levels in all three groups. In contrast, no significant correlation of PKM2 protein expression was associated with *ERCC1*, *NEDD9*, and *TS* mRNA expression. Finally, overexpression of *PKM2* was significantly recorded in tumors with microsatellite stability (MSS) status and those with infiltrated regional lymph nodes in group-A, but not in groups B and C.

The present study enriched previous knowledge by demonstrating that high *PKM2* mRNA levels were strongly associated with adverse outcome of CC patients treated with FOLFOX. In the same line of evidence, previous studies from our laboratory demonstrated that low *PKM2* mRNA levels were associated with better survival rates in NSCLC [17] and low *PKM2* expression attained significantly better PFS and OS in SCLC patients treated with platinum-based chemotherapy [16], respectively.

The major advantage of this study is that the predictive significance of *PKM* mRNA expression in operable CC was validated in an independent large cohort of patients mCRC treated with the same chemotherapy and in a control group of patients treated with a different regimen. Besides the robust results for *PKM2* mRNA expression, the findings should be interpreted with caution and mainly as a hypothesis generated results. Consequently, it remains a challenge that has to be investigated using in vitro and in vivo models to elucidate the molecular mechanisms underlying PKM2 regulation, either transcriptional or post-transcriptional, which could modulate anticancer-drug cytotoxicity. The analysis of the predictive value of *PKM2* mRNA expression treated with CAPOX or FOLFOX in the IDEA–HORG study [35] is underway in order to validate the results of the current study in a large cohort of patients treated in the context of the clinical trial.

## 4. Materials and Methods

Formalin-fixed, paraffin-embedded (FFPE) tissues from 262 consecutive patients with histologically confirmed stage II/III CC and 118 metastatic CRC (mCRC) patients treated with oxaliplatin-based chemotherapy were collected and analyzed. In addition, 104 mCRC patients treated with FOLFIRI were used as a control group. Furthermore, 51 matching normal mucosa biopsies from the above 262 patients and 50 benign hyperplastic polyps and adenomas were included in the analysis. The study was approved by the Ethics and Scientific Committees of the University General Hospital of Heraklion (No: 2058) and patients gave their written informed consent.

FFPE tumor sections were examined by a pathologist (MT) in order to identify the most tumor-enriched areas for dissection. In the case of samples with <80% tumour cells, an Eppendorf piezoelectric microdissector was used to procure only malignant cells. DNA and RNA extraction was performed as previously described [36,37]. *KRAS* exon 2 (codon 12 and 13) and *BRAF^V600E^* mutation was performed as previously reported [37]. For MSI testing, DNA of each tumor and that of a normal patient was analyzed using the Promega MSI Analysis System according to the manufacturer’s instructions. Microsatellite status was defined in accordance with the Bethesda guidelines [38].

cDNA synthesis from total RNA and RT-PCR was performed as described previously [36]. The primers and probes for both housekeeping and target genes are shown in Table S1 and were designed using the PrimerExpress 2.0 Software (Applied Biosystems, Foster City, CA, USA) according to the RefSeq NM_002654 and NM_002467.4.

Disease-free survival (DFS) measured the length of time after the date of surgery to the first documented metastatic disease, second primary CC, or death from any cause. Progression free survival (PFS) was estimated from the data of first-line treatment initiation to documented disease progression or death. Overall survival (OS) was defined the length from the surgery date to the date of death. The Kaplan–Meier survival curves were used to evaluate the impact of various variables in the OS of patients. A Cox proportional hazards model was used to assess the effect of the assessed parameters on death events. These factors were then included in a multivariate Cox proportional hazards regression model with a stepwise procedure (both forward and backward) to evaluate the independent significance of different variables on survival and time to progression, as previously described [17].A *p*-value < 0.05 was used for significance. All the laboratory analyses were performed blindly to the clinical data. Associations between *KRAS*, *BRAF* mutation status and MSI status, and *PKM2* mRNA expression with baseline characteristics were estimated using the Fisher’s exact test for categorical variables or logistic regression for continuous variables [37,39].

## 5. Conclusions

The current study provides evidence that patients with operable colon cancer treated with FOLFOX with high expression of PKM2 mRNA presented lower PFS and OS. In addition, it is found that lower PFS and OS were detected in a cohort of mCRC pts treated with FOLFOX. On the other hand, no significant correlations of high PKM2 mRNA and PFS/OS detected in the metastatic group of patients who received FOLFIRI. Finally, yet importantly, we reported findings showing significantly lower levels of PKM2 expression, associated in adenoma with LG dysplasia or in normal mucosa in contrast with HG dysplasia adenomas or CC.

## Figures and Tables

**Table 1:** Characteristics and pathological features of the patients.

Pts	Group A (262)		Group B (118)		Group C (104)	
	N	%	N	%	N	%
**Median Age (Range)**	67 (33–75)		65 (35–84)		65 (41–79)	
≤70 years	162	62	77	65	78	73
>70 years	100	38	41	35	26	27
**Gender**						
Male	152	58	76	64	66	64
Female	110	42	42	36	38	36
**Performance Status (ECOG) ^#^**						
0	196	75	110	93	98	94
1	66	25	8	7	6	6
**Stage**						
IIa	90	34				
IIb	14	6				
IIIa	19	7				
IIIb	81	31				
IIIc	58	22				
IV			118	100	104	100
**Tumor grade**						
low	160	61	69	59	60	58
high	102	39	49	41	44	42
**Mucinous**						
Yes	59	23				
No	203	77				
**Obstruction**	27	10				
**Perforation**	40	15				
**Location**						
Right-sided	91	35	36	31	26	25
Left-sided	171	65	82	69	78	75
**Regimen**						
CAPOX	171	65				
FOLFOX	91	35				
FOLFOX/CAPOX			46	39		
FOLFOX/CAPOX + Bevacizumab			45	38		
FOLFOX/CAPOX + Cetuximab			27	23		
FOLFIRI					18	17
FOLFIRI + Bevacizumab					86	83
***BRAF^V600E^* status**						
Wild type (WT)	230	87.8	110	93	97	94
Mutant	13	5.0	8	7	7	6
Failed	19	7.2				
***KRAS* exon 2 mutation**						
WT	169	64.5	76	64	69	66
Mutant	82	31.3	42	36	35	34
Failed	11	4.2				
**MMR Status**						
Proficient	200	76.3	Not Done		Not Done	
Deficient	35	13.4	Not Done		Not Done	
Failed	27	10.3				
**Median No of Retrieved Lymph Nodes (min–max)**	15 (6–108)					
**Median No of +ve Lymph Nodes** **(min–max)**	1 (0–18)					
**Median Number of metastatic sites**			1 (1–4)		1 (1–5)	
**Metastatic disease**						
Synchronous			44	37	30	29
Metachronous			74	63	74	71

**Table 2:** M2 isoform of pyruvate kinase (PKM2) mRNA expression.

PKM2 mRNA Expression.									
	(*n* = 258)			(*n* = 118)			(*n* = 104)		
	Group A			Group B			Group C		
	Low (*n* = 129)	High (*n* = 129)	*p* value	Low (*n* = 117)	High (*n* = 137)	*p* value	Low (*n* = 117)	High (*n* = 137)	*p* value
**Patients enrolled**	N (%)	N (%)		N (%)	N (%)		N (%)	N (%)	
**Age Median (min–max)**	67 (33–75)	67 (37–75)	0.147 ^#^	65 (35–81)	65 (39–84)	0.564 ^#^	65 (44–76)	64 (41–79)	0.218 ^#^
**Age group**									
≤70	72 (45.3)	87 (54.7)	0.08 ^@^	52 (67.5)	25 (32.5)	0.499 ^@^	47 (60.2)	31 (39.8)	0.614 ^@^
>70	57 (57.5)	42(42.5)		28 (68.2)	13 (31.8)		15 (57.7)	11 (42.3)	
**Gender**									
Male	75 (51.0)	72(49.0)	0.669 *	49 (64.5)	27 (35.5)	0.411 *	35 (53.0)	31 (47)	0.571 *
Female	52 (47.7)	57(52.3)		31 (73.8)	11 (26.2)		21 (55.3)	17 (34.7)	
**Lymph Node Status**									
N0	63(61.8)	39 (38.2)	0.018 *						
N1–2	64 (41.8)	89 (58.2)							
**Tumor Location**									
Right	55 (60.4)	36 (39.6)	0.011 *	24 (66.7)	12 (33.3)	0.003 *	16 (61.5)	10 (38.5)	0.001 *
Left	74 (44.3)	93 (56.7)		39 (47.6)	43 (52.4)		29 (37.2)	49 (62.8)	
**Grade**									
Low grade	117 (74.5)	41 (25.5)	0.001 *	51 (73.9)	18 (26.1)	0.017 *	43 (71.7)	17 (18.3)	0.021 *
High grade	12 (12.0)	88 (88.0)		8 (16.3)	41 (83.7)		7 (15.8)	37 (84.2)	
**Mucinous**									
Yes	40 (68.9)	18 (31.1)	0.001 *						
No	89 (44.5)	111 (55.5)							
***KRAS* exon 2 status**									
Wild type	87 (51.5)	83 (48.5)	0.87	47 (61.8)	29 (38.2)	0.009 *	46 (66.7)	23 (33.3)	0.026 *
Mutant	40 (48.8)	42 (51.2)		6 (14.3)	36 (85.7)		9 (25.7)	26 (74.3)	
UKNOWN	2	4		0	0				
***BRAF^V600E^* status**									
Wild type	112 (48.1)	117 (51.9)	0.109	74 (67.3)	36 (32.7)	0.041 *	58 (59.8)	39 (40.2)	0.05 *
Mutant	8 (61.5)	5 (38.5)		1 (12.5)	7 (87.5)		1 (16.7)	6 (83.3)	
UKNOWN	9	7		0	0		0	0	
**MMR Status**									
Proficient	88 (44)	108 (56)	<0.001						
Deficient	29 (82.8)	6 (17.2)							
UKNOWN	12	15							

^#^ Mann–Whitney test; * Pearson Chi-square; ^@^ Fisher’s exact.

**Table 3:** Univariate analysis for progression free survival (PFS) and overall survival (OS). HR, hazard ratio; CI, confidence interval.

Pts	Group A		Group B		Group C	
Feature	HR ^#^ (95% CI ^^^)	*p* Value	HR ^#^ (95% CI ^^^)	*p* Value	HR ^#^ (95% CI ^^^)	*p* Value
**PFS**						
Age (>70 y vs. ≤70 y)	1.03 (0.64–1.67)	0.887	1.17 (0.89–1.32)	0.214	1.21 (0.94–1.41)	0.167
Gender (Men vs. Women)	1.18 (0.92–1.80)	0.112	1.07 (0.61–1.18)	0.816	1.04 (0.63–1.14)	0.883
Stage (III vs. II)	1.80(1.61–2.10)	0.023				
Tumor Location (Right vs. Left)	1.13 (0.68–1.87)	0.234	1.34 (1.09–2.09)	0.037	1.29 (1.06–1.99)	0.043
Grade (High vs. Low)	1.86 (1.18–3.08)	0.722	1.16 (0.84–1.23)	0.304	1.21 (0.79–1.94)	0.682
*PKM2* mRNA expression (High vs. Low)	1.82 (1.21–2.96)	0.003	1.91 (1.34–2.99)	0.002	1.06 (0.65–1.31)	0.769
*KRAS* exon2 mutation (Mutants. ^&^ vs. wild type ^@^)	1.76 (1.09–3.10)	0.050	1.85 (1.16–2.85)	0.047	1.80 (1.09–2.91)	0.046
*BRAF^V600E^* mutation (Mut. ^&^ vs. wt ^@^)	1.97 (1.79–2.50)	0.001	3.02 (2.46–5.73)	0.001	2.88 (1.93–50.8)	0.001
MMR status (Proficient vs. Deficient)	1.73 (1.29–3.51)	0.025				
**OS**						
Age (>70 y vs. ≤70 y)	1.01 (0.98–1.04)	0.781	1.22 (0.94–1.38)	0.189	1.24 (0.96–1.44)	0.118
Gender (Men vs. Women)	1.21 (0.94–1.84)	0.106	1.11 (0.72–1.24)	0.603	1.17 (0.77–1.32)	0.712
Stage (III vs. II)	1.64 (1.45–2. 01)	0.030				
Tumor Location (Right vs. Left)	1.02 (0.56–1.85)	0.906	1.44 (1.18–2.31)	0.018	1.39 (1.23–2.08)	0.023
Grade (High vs. Low)	1.11 (0.57–2.21)	0.781	1.09 (0.88–1.21)	0.446	1.14 (0.8–1.91)	0.588
*PKM2* mRNA expression (High vs. Low)	1.84 (1.29–3.26)	0.002	2.12 (1.51–3.17)	0.002	1.04 (0.58–1.24)	0.811
*KRAS* exon2 mutation (Mut. ^&^ vs. wt ^@^)	1.20 (0.61–2.33)	0.113	1.56 (0.98–1.94)	0.077	1.49 (0.96–2.03)	0.102
*BRAF^V600E^* mutation (Mut. ^&^ vs. wt ^@^)	1.62 (1.14–2.31)	0.007	3.02 (2.46–5.73)	0.001	2.88 (1.93–50.8)	0.001
MMR status (Proficient vs. Deficient)	1.38 (1.04–2.71)	0.036				

^#^ Hazard ratio; ^^^ confidence interval; ^&^ mutant; ^@^ wild type.

**Table 4:** Multivariate analysis for disease free survival (DFS, group-A), progression free survival (PFS, groups B and C), and median overall survival.

Feature	Group A		Group B		Group C	
	HR ^#^ (95% CI ^^^)	*p* Value	HR ^#^ (95% CI ^^^)	*p* Value	HR ^#^ (95% CI ^^^)	*p* Value
**DFS/PFS**						
Stage (III vs. II)	1.27 (1.03–1.76)	0.046				
Tumor Location (Right vs. Left)			1.17 (0.91–1.88)	0.121	1.12 (0.88–1.69)	0.198
PKM2 mRNA expression (High vs. Low)	1.88 (1.37–2.99)	0.002	1.94 (1.38–3.32)	0.001	1.08 (0.66–1.33)	0.517
KRAS exon2 mutation (Mut. ^&^ vs. wt ^@^)	1.35 (0.92–1.91)	0.103	1.31 (0.91–2.06)	0.197	1.29 (0.86–1.91)	0.267
*BRAF^V600E^* mutation (Mut. ^&^ vs. wt ^@^)	1.98 (1.64–2.67)	0.001	3.61 (2.67–5.81)	<0.001	3.43 (2.58–5.79)	<0.001
MMR status (Proficient vs. Deficient)	1.76 (1.31–3.44)	0.021				
**OS** (overall survival)						
Stage (III vs. II)	1.33 (1.09–1.88)	0.039				
Tumor Location (Right vs. Left)			1.25 (0.98–2.07)	0.081	1.118 (0.93–1.88)	0.092
PKM2 mRNA expression (High vs. Low)	1.91 (1.45–2.97)	0.001	1.99 (1.49–3.41)	0.001	1.03 (0.59–1.39)	0.612
KRAS exon2 mutation (Mut. ^&^ vs. wt ^@^)	1.35 (0.92–1.91)	0.103	1.31 (0.91–2.06)	0.197	1.29 (0.86–1.91)	0.267
*BRAF^V600E^* mutation (Mut. ^&^ vs. wt ^@^)	2.12 (1.69–2.91)	<0.001	3.78 (2.81–5.66)	<0.001	3.64 (2.66–5.61)	<0.001
MMR (Mismatch Repair System) status (Proficient vs. Deficient)	1.89 (1.47–3.52)	0.002				

^#^ Hazard ratio; ^^^ confidence interval; ^&^ mutant; ^@^ wild type.

## Supplementary Materials

The following are available online at https://www.mdpi.com/2072-6694/12/8/2058/s1, Table S1: Sequence of the primers and probes of all reference and target genes.
