# Supplementary material for: PKM2 Expression as Biomarker for Resistance to Oxaliplatin-Based Chemotherapy in Colorectal Cancer
Source: Cancers (Basel). 2020 Jul 25;12(8):2058. doi: 10.3390/cancers12082058 (PMC7465271; doi:10.3390/cancers12082058)
Supplement: Supplementary file 1 [file cancers-12-02058-s001.pdf]

cancers

MDPI

# Supplementary Material: PKM2 Expression as Biomarker for Resistance to Oxaliplatin-Based Chemotherapy in Colorectal Cancer

**Maria Sfakianaki, Chara Papadaki, Maria Tzardi, Maria Trypaki, Stavroula Manolakou, Ippokratis Messaritakis, Zenia Saridaki, Elias Athanasakis, Dimitrios Mavroudis, John Tsiaoussis, Nikolaos Gouvas and John Souglakos**

**Table S1: Sequence of the primers and probes of all reference and target genes.**

| Gene | Forward Primer | 5′-labeled (6FAM) Probe | Reverse Primer |
|---|---|---|---|
| *c-MYC* | 5′GAGCCCCTGGTGCTCCAT 3′ | 5′AGGAGACACCGCCCAC-3′ | 5′TCA TCTTCTTGTTCCTCCTCAGAGT-3′ |
| *ERCC1* | 5′GGGAATTTGGCGACG TAATTC-3′ | 5′CACAGGTGCTCTGGC CCAGCACATA-3′ | 5′GCGGAGGCTGAGGAACAG-3′ |
| *NEDD9* | 5′-GGGTAAAAAGGTGATAACCCCCGT-3′ | 5′-CAAGGGCCTTAT ATGAC-3′ | 5′TGCTGATGAGGGAGGGATGTCGT-3 |
| *TS* | 5′AATCACATCGAGCCA CTG AAA AT-3′ | 5′CAG CTT CAG CGA GAA C-3′ | 5′AATCCTGAGCTTTGGGAAAGG-3′ |
| *PKM2* | 5′ GCCATAATCGTCCTCACC 3′ | 5′ CAGGTCTGCTCACCAGG 3′ | 5′ GCACGTGGGCGGTATCTG 3′ |
| *B-Actin* | 5′ GGCACCCAGCACAATGAAG 3′ | 5′ TCAAGATCATTGCTCCTCCTGAGCGC 3′ | 5′ GCCGATCCACACGGAGTACT 3′ |
| *PGK1* | 5′ GGCTGGATGGGCTTGGA 3′ | 5′ TGTGGTCCTGAAAGCAGCAAGAAGTATG 3′ | 5′ TCTGCTTAGCCCGAGTGACA3′ |
